# Multiple cardiotoxicities during osimertinib therapy

**DOI:** 10.1177/10781552231164301

**Published:** 2023-03-21

**Authors:** Hasan Kobat, Michael Davidson, Islam Elkonaissi, Emma Foreman, Shereen Nabhani-Gebara

**Affiliations:** ^1^ Department of Pharmacy, School of Life Sciences, Pharmacy and Chemistry, Kingston University London, Kingston Upon Thames, UK; ^2^ Lung Unit, 4970 The Royal Marsden NHS Foundation Trust, London, UK; ^3^ 591854 Sheikh Shakhbout Medical City, Abu Dhabi, UAE; ^4^ Pharmacy Department, 4970 The Royal Marsden NHS Foundation Trust, London, UK

**Keywords:** Lung cancer, anti-cancer treatment, drug safety, cardiotoxicity, case report

## Abstract

**Introduction:**

The tyrosine-kinase inhibitor osimertinib is an oral anti-cancer agent that is used for the treatment of patients with metastatic non-small cell lung cancer harbouring sensitising *EGFR* mutations. Patients receiving osimertinib are at higher risk of developing cardiac toxicity, and here we present the case of a 72-year-old male who developed multiple cardiotoxicities during therapy (i.e. QTc prolongation, atrial fibrillation, heart failure).

**Case Report:**

A 72-year-old white British, ex-smoker male patient was admitted to our cancer centre with adenocarcinoma of the lung. Afatinib, gefitinib, osimertinib, and carboplatin plus pemetrexed chemotherapy were the treatments he received. At the 15th month of osimertinib therapy, the patient developed QTc prolongation. Two weeks after the first incidence of QTc prolongation, electrocardiography showed rate-controlled atrial fibrillation. In addition to his atrial fibrillation, echocardiography revealed severely impaired left ventricular systolic function (left ventricular ejection fraction: 30%).

**Management and Outcomes:**

Baseline to osimertinib, an electrocardiography investigation was carried out as per the protocol. Baseline drug history was reviewed and rosuvastatin was discontinued before initiating osimertinib as both drugs contribute to QTc prolongation. Dabigatran, bisoprolol, and digoxin were started for the treatment of atrial fibrillation. Ramipril and spironolactone were prescribed for the treatment of heart failure but osimertinib continued uneventfully. The patient died of non-small cell lung cancer.

**Discussion:**

Recommendations for practical and clinically relevant baseline and on-treatment assessments are considered which may reduce the risk of cardiac toxicity during osimertinib therapy. These include baseline cardiac risk stratification, consideration of concomitant medications that may result in additive cardiac risk, and use of electrocardiography and echocardiography surveillance.

## Introduction

Lung cancer is the second most common cancer accounting for 11.4% of all cancer cases and the leading cause of cancer-related death, globally.^1^ Nearly 85% of the lung cancer cases are of the non-small-cell lung cancer (NSCLC) subtype, and the 5-year survival rate is <15%.^2^ For a biologically defined subgroup of NSCLCs harbouring a targetable genetic mutation, tyrosine kinase inhibitor (TKI) drugs have resulted in durable survival benefits.^3–9^ Longer exposure to these drugs can result in development of both acute and long-term toxicities, and cardiotoxicity is increasingly recognised as a relatively common and challenging side effect.

Cardiotoxicities have been associated with a number of TKIs used in the treatment of lung cancer and can cause both treatment disruption and diminish quality of life.^10–13^ Cardiotoxic events that are attributed to the use of these TKIs include, but are not limited to, QTc prolongation, left ventricular dysfunction, heart failure, arrhythmia, ischaemia, myocardial infarction and fluctuations in blood pressure.^13^ The true incidence of cardiotoxicity associated with TKIs is potentially greater than that reported in clinical trial data due to a lack of systematic screening, risk assessment and monitoring.

Osimertinib (TagrissoTM, AZD9291) was approved in 2015 by the U.S. Food and Drug Administration (FDA) for the treatment of epidermal growth factor receptor (*EGFR*) *T790 M* mutation-positive metastatic NSCLC with a starting dose of 80 mg once a day orally.^14^ The FDA Adverse Events Reporting System lists cardiac failure, atrial fibrillation, QT prolongation, myocardial infarction and pericardial effusion as potential toxicities with additional case study reports of various cardiotoxicities.^10, 15–18^

Here we report the case of a patient with NSCLC who developed multiple cardiotoxicities during osimertinib therapy. Optimal management strategies for the prevention and early diagnosis of cardiotoxicity are also discussed.

## Case report

### Patient background

The patient presented in this case report was deceased. Exhaustive attempts have been made to seek consent from the next of kin but failed to contact them. The paper has been sufficiently anonymised not to cause harm to the patient and family. A 72-year-old white British, ex-smoker male patient was referred to The Royal Marsden NHS Foundation Trust in 2015 with metastatic adenocarcinoma of the lung with a history of type two diabetes (for 17 years), hypercholesterolaemia (for 12 years), hypertension (for 12 years), oesophagitis (for 20 years) and gastritis (for 20 years). He had excellent current performance status with Eastern Cooperative Oncology Group performance status of 0, leading an active life. Drug history included bendroflumethiazide (2.5 mg, 10 years), amlodipine (10 mg, 5 years), ramipril (10 mg, 9 years), metformin (1500 mg, 17 years), glimepiride (2 mg, 9 years), lansoprazole (15 mg, 20 years), tamsulosin (400 microg, 5 years), aspirin (75 mg, 7 years), cetirizine (10 mg, 1 year), rosuvastatin (10 mg, 2 years) and vitamin B supplementation. Baseline systolic/diastolic blood pressure was 121/69 mm/Hg and heart rate being 73 beats per minute.

Computerised tomography (CT) showed a 3.5 cm lesion in the left hilum with left aortopulmonary window nodes, right adrenal mass, and low attenuation liver lesions. Subsequent investigation revealed stage IV lung adenocarcinoma found to harbour a sensitising *l858R EGFR* mutation.

### Anti-cancer treatment

**Figure 1.**
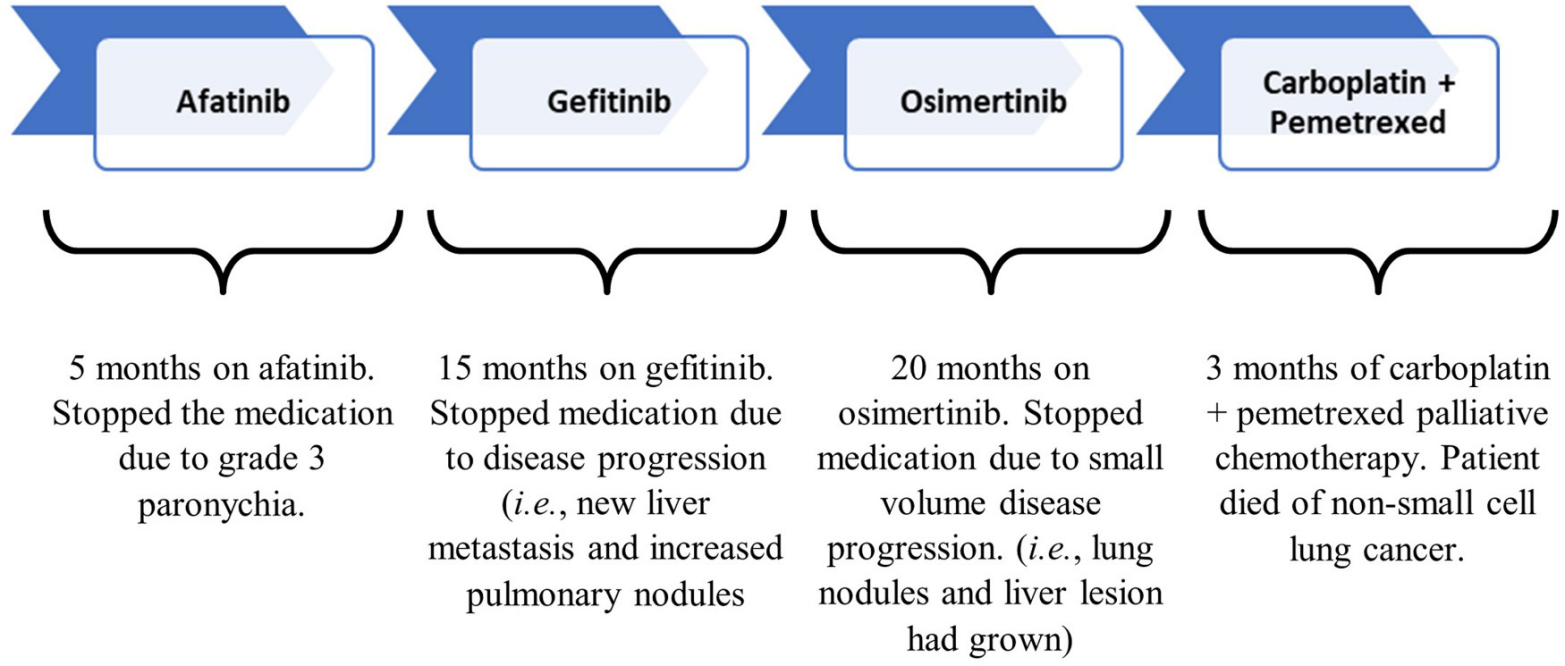
Demonstration of treatment with anti-cancer agents, exposure times and the reasons for regimen changes.

First-line monotherapy of afatinib was initiated which was followed by gefitinib, osimertinib and carboplatin + pemetrexed therapy. The patient was on afatinib for 5 months before being discontinued because of grade 3 paronychia, and a skin infection around his toenails.^19^ The patient was given gefitinib for 15 months until discontinuation due to new liver metastasis and increased pulmonary nodules. He then received osimertinib for 20 months which was stopped because of small volume progression of the lung nodules and liver lesion. Palliative chemotherapy, the last treatment, with carboplatin and pemetrexed was initiated. Three months after commencing carboplatin + pemetrexed therapy, a CT showed that the primary tumour was stable, with 25% shrinkage in the liver lesion (Figure 1). CT showed disease progression onwards and the patient died of NSCLC. Overall survival, from the date of diagnosis until death, was 4 years and 8 months.

### Development of cardiotoxicities

#### QTc prolongation

**Figure 2.**
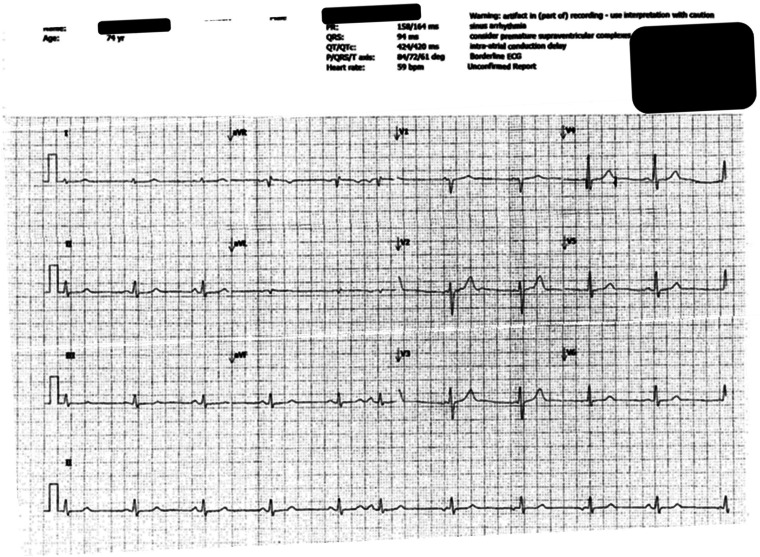
Baseline electrocardiography (ECG) showing normal sinus rhythm prior to oslimertinib therapy.

Baseline to osimertinib, electrocardiography (ECG) showed normal sinus rhythm with QTc interval being 420 ms (Figure 2). After a medicines review by a pharmacist, rosuvastatin was stopped before starting osimertinib as it can contribute to QTc prolongation as osimertinib does.^20^ At the 15th month of osimertinib therapy, the patient was admitted to the hospital with shortness of breath. The oncology team was informed that the patient developed QTc prolongation (>480 ms).

#### Atrial fibrillation and heart failure

**Figure 3.**
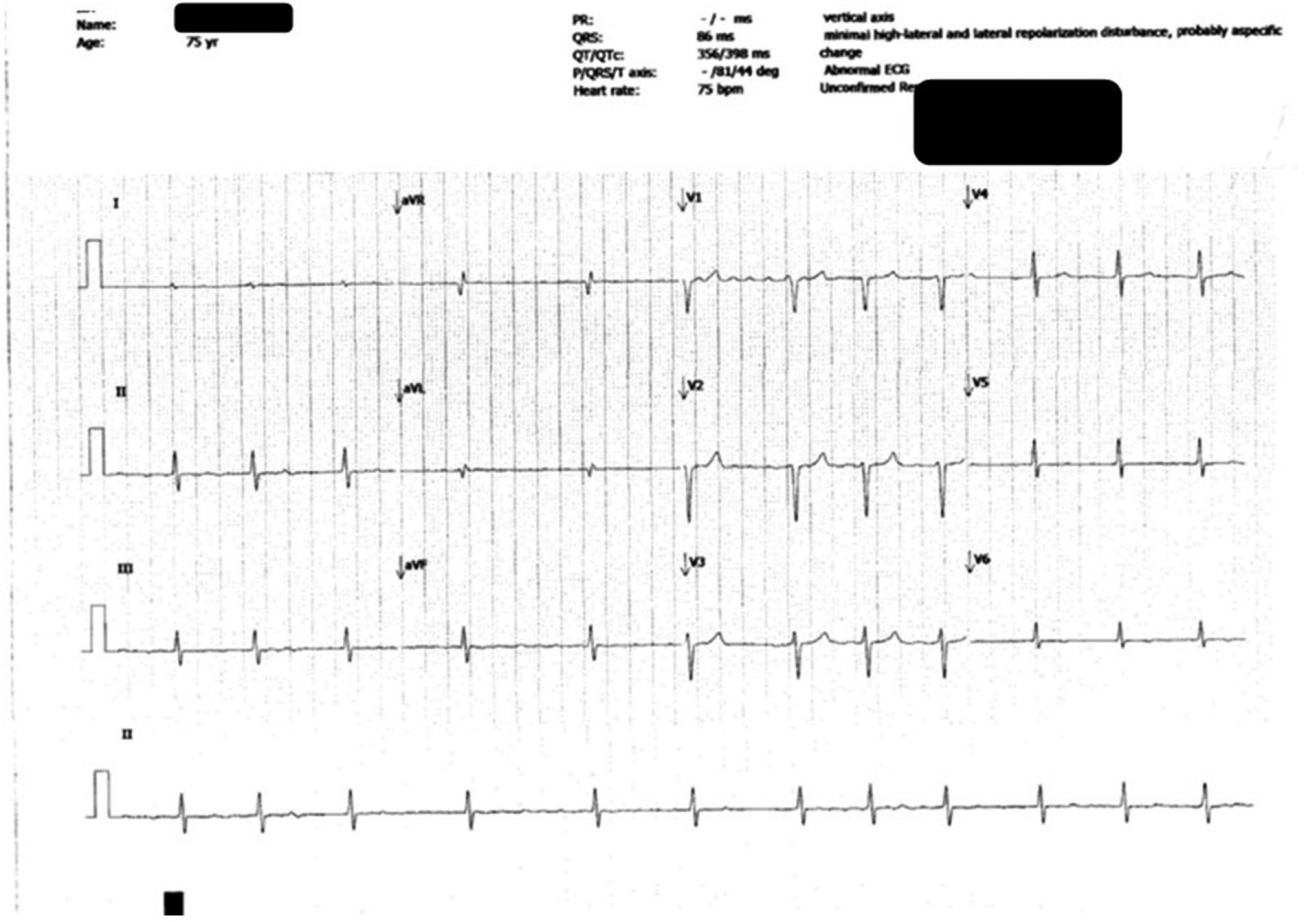
Electrocardiography (ECG) showing cardiotoxicity (i.e. atrial fibrillation) at the 15th month of osimertinib therapy.

Two weeks after the first incidence of QTc prolongation, ECG showed rate-controlled atrial fibrillation at a rate of 75 beats per minute with normalised QTc interval, 398 ms (Figure 3). Dabigatran, bisoprolol and digoxin were started for the treatment of atrial fibrillation.

In addition to his atrial fibrillation, echocardiography revealed severely impaired left ventricular systolic function (left ventricular ejection fraction: 30%). Ramipril and spironolactone were prescribed for the treatment of heart failure.

**Figure 4.**
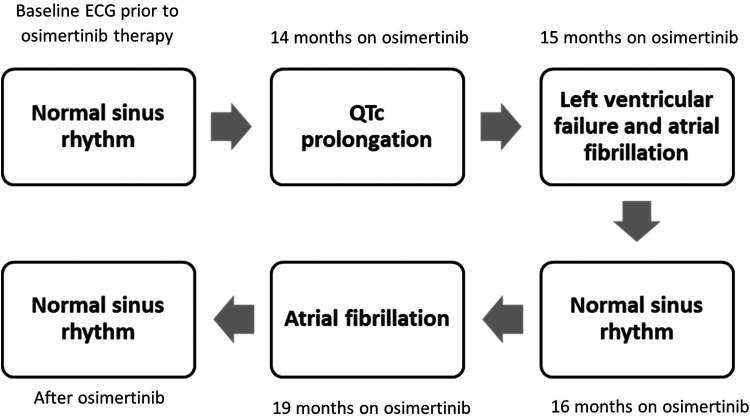
Demonstration of cardiotoxicities during osimertinib therapy. Baseline electrocardiography (ECG) showed normal sinus rhythm. At the 14th month of the osimertinib treatment ECG demonstrated QTc prolongation. A month later, ECG showed atrial fibrillation with normal QTc interval. At the same time, echocardiography revealed left ventricular ejection fraction of 30%. A month after the onset of heart failure, the patient demonstrated normal sinus rhythm on ECG. At the 19th month of osimertinib treatment, patient showed atrial fibrillation on ECG which is then recovered after osimertinib was stopped.

After optimisation of cardiac management, osimertinib continued uneventfully. Naranjo algorithm score of 7 was obtained via online tool that indicates cardiotoxicity event was a ‘probable’ adverse drug reaction (see Supplemental Appendix).^21^ The full history of cardiotoxicity incidences are shown in Figure 4.

## Discussion

### Cardiotoxicities during osimertinib

According to our ongoing internal audit of cardiac toxicity on TKIs, this patient is the only one who developed three discrete cardiotoxicities during osimertinib, namely of QTc prolongation, atrial fibrillation and heart failure. According to a phase 3 trial of osimertinib, 10.4% of the patients developed QTc prolongation in the osimertinib subgroup whereas the incidence was only 4% in the control group.^22^ Another phase 3 clinical trial also demonstrated QTc prolongation in 10 out of 279 (3.6%) patients who were treated with osimertinib.^5^ In our case, patient's regular medicines were also reviewed, and QT-prolonging drugs were stopped before starting osimertinib, allowing the QTc prolongation potential to be attributed to osimertinib.

Atrial fibrillation has also been associated with osimertinib in phase 3 clinical trials and in real-world practice.^10^ Analysis of reported adverse events in patients receiving osimertinib found that heart failure, atrial fibrillation and QT prolongation were more frequently associated with osimertinib when compared with other TKIs.^10^

According to the FLAURA trial, 12 out of 279 patients (4.3%) developed cardiac failure of whom 10 cases were associated with impaired systolic functions.^22^ In our case, the patient had also developed heart failure associated with systolic dysfunction. The patient did not have any pre-existing heart disease prior to osimertinib and was not on any medicines with the potential to cause a decrease in left ventricular ejection fraction to a level of 30%. Osimertinib is therefore believed to be responsible for his heart failure.

### Management strategies

#### Baseline and serial monitoring with ECG

Cardio-oncology is an emerging field that involves the management of oncology patients from a cardiology perspective. Its interprofessional and multidisciplinary nature requires strong collaboration among oncology and cardiology teams. Several principles including prevention, serial monitoring, early diagnosis, and early treatment are significant elements of cardio-oncology to protect patients from unforeseen cardiotoxicity.^23^ QTc prolongation is among the most frequently seen cardiac adverse events due to osimertinib. This is usually asymptomatic and requires serial monitoring with ECG.^24^ Serial ECG monitoring is listed in prescribing information but without suggesting its frequency.^14^ Monthly ECGs during osimertinib therapy should be a part of clinical practice to detect asymptomatic cardiotoxicity. We would advocate that at baseline and before every cycle of osimertinib and other anti-cancer agents that may cause electrical activity changes of the heart, ECG assessment should be undertaken.

#### Echocardiography as part of the management strategy

Even though left ventricular dysfunction is a rarer toxicity it can be life-threatening.^25^ A decrease in left ventricular ejection fraction is reported in trial data and real-world studies. According to a study by *Kunimasa* et al.,^26^ 58 out of 183 patients who received osimertinib were assessed with echocardiography at baseline and during treatment. And 8.7% of patients experienced a decline in left ventricular ejection fraction ≥10%. Echocardiography assessment at baseline and serially throughout treatment should ideally be part of the routine monitoring of patients receiving osimertinib, but this is neither easily applicable nor cost-effective. Echocardiography evaluations are not as practical as ECG assessments, and a comprehensive plan within each institution should be made to sustain the most efficient level of monitoring. The number of institutions carrying out echocardiography baseline to osimertinib is low globally. Instead, risk factor stratification at baseline may guide healthcare professionals in determination of high-risk patient who will benefit the most from the addition of echocardiography to their monitoring schedule.

#### Baseline review of drug history and risk factors

Reviewing drug–drug interactions is critical prior to starting potentially cardiotoxic anti-cancer treatment. Drugs that can potentially alter the electrical activity of the heart, most importantly QTc prolonging agents, should be reconsidered if possible as the risk of QTc prolongation increases with concomitant use. In this patient, rosuvastatin which is known to trigger the QTc prolongation was stopped. Reviewing medicines at baseline and replacing or stopping those which cause QTc prolongation decreases the risk of cardiotoxicity. If such drugs are essential, closer monitoring is required.

Pre-existing conditions such as hypertension, diabetes and hypercholesterolaemia are risk factors for cardiovascular disease and the risk of cardiotoxicity development is increased in patients with such cardiovascular comorbidities. Patients demonstrating baseline conditions that can promote cardiotoxicity should be followed up closely at baseline, during, and after treatment with osimertinib.

#### Improving the cardio-oncology practice

At our institution and in the United Kingdom, cardio-oncology screening is not standardised for the treatment of NSCLC. At the moment, cardio-oncology care in the United Kingdom is reactive where patients are referred to cardiology in light of emerging cardiotoxicity. A proactive approach of periodic monitoring is needed to optimise cardio-oncology care and overall outcomes. Improving cardio-oncology practice at oncology centres needs cardiology collaborations. Cancer centres that established successful cardio-oncology care have cardiologists who are specialised in cardio-oncology and work only for a cancer centre. The majority of UK cancer centres do not have in house cardiologists. This may slow down and restrict the improvements in care as well as lead to miscommunications. Cardiotoxicity profiles and trends of TKIs used in the treatment of NSCLC have not yet been well established. However, our team has been running studies to investigate the true incidence and trends of cardiotoxicity in a large sample of patients with lung cancer. Facilitating real-world studies in cardio-oncology is vital in standardising cardio-oncology care. Understanding the risk groups and true incidence of cardiotoxicity secondary to targeted treatments will improve the standardisation of care, however, requires a proactive cardio-oncology approach. Increased cardio-oncology research may reveal the high-risk groups, which may contribute to lower the cardiotoxicity risk and improving patient outcomes.

## Conclusion

In this case report, we present a patient who developed multiple cardiotoxicities during osimertinib therapy. Individual cardiac risk assessments should be a part of routine clinical practice to identify patients at high risk. Cardiovascular examinations using ECG and echocardiography at baseline, during, and after therapy, as well as a review of drugs and medical history, should be integrated and evaluated holistically to prevent, identify, and treat cardiotoxicity early.

## Supplemental Material

sj-docx-1-opp-10.1177_10781552231164301 - Supplemental material for Multiple cardiotoxicities during osimertinib therapy Supplemental material, sj-docx-1-opp-10.1177_10781552231164301 for Multiple cardiotoxicities during osimertinib therapy by Hasan Kobat, Michael Davidson, Islam Elkonaissi, Emma Foreman and Shereen Nabhani-Gebara in Journal of Oncology Pharmacy Practice
