# Supplementary material for: Multiple cardiotoxicities during osimertinib therapy
Source: J Oncol Pharm Pract. 2023 Mar 21;32(2):392–8. doi: 10.1177/10781552231164301 (PMC13018250; doi:10.1177/10781552231164301)
Supplement: sj-docx-1-opp-10.1177_10781552231164301 - Supplemental material for Multiple cardiotoxicities during osimertinib therapy [file sj-docx-1-opp-10.1177_10781552231164301.docx]

22/11/2022, 10:59 REPORT_14254(22.11.2022-10:58)

Naranjo Algorithm

2022-11-22 11:58:09

1. Are there previous conclusive reports on this reaction? Yes [+1]
2. Did adverse event appear after the suspected drug was given? Yes [+2]
3. Did the adverse reaction improve when the drug was discontinued or a specific antagonist was given? Yes [+1]
4. Did the adverse reaction appear when the drug was readministered? Do not know or not done [0]
5. Are there alternative causes that could have caused the reaction? No [+2]
6. Did the reaction reappear when a placebo was given? Do not know or not done [0]
7. Was the drug detected in any body fluid in toxic concentrations? Do not know or not done [0]
8. Was the reaction more severe when the dose was increased, or less severe when the dose was decreased? Do not know or not done [0]
9. Did the patient have a similar reaction to the same or similar drugs in any previous exposure? No [0]
10. Was the adverse event confirmed by any objective evidence? Yes [+1]

→ Naranjo Score 7

→ **Adverse Drug Reaction** PROBABLE

*Cut-off points include:*

*≤0 DOUBTFUL ADR 1-4 POSSIBLE ADR*

*5-8 PROBABLE ADR*

*≥9 DEFINITE ADR*

tools.farmacologiaclinica.info/printanswers.php?sid=14254 1/1
